# Investigation of brain tissue infiltration by medulloblastoma cells in an *ex vivo* model

**DOI:** 10.1038/s41598-017-05573-w

**Published:** 2017-07-13

**Authors:** Anuja Neve, Karthiga Santhana Kumar, Dimitra Tripolitsioti, Michael A. Grotzer, Martin Baumgartner

**Affiliations:** 1 Children’s Research Centre, Oncology, University Children’s Hospital, August-Forel Strasse 1, CH-8008 Zürich, Switzerland; 2 Department of Oncology, University Children’s Hospital Zürich, Steinwiesstrasse 75, CH-8032 Zürich, Switzerland

## Abstract

Medulloblastoma (MB) is a paediatric cancer of the cerebellum that can develop cerebellar and leptomeningeal metastases. Local brain tissue infiltration, the underlying cause of metastasis and relapse, remains unexplored. We developed a novel approach to investigate tissue infiltration of MB using organotypic cerebellum slice culture (OCSC). We show that cellular and structural components of cerebellar tissue in OCSCs are maintained for up to 30 days *ex vivo*, and that OCSCs foster tumour growth and cell proliferation. Using cell-based models of sonic hedgehog (SHH) and group 3 (G3) MB, we quantified tumour growth and infiltration and determined the morphological characteristics of the infiltrating cells. We observed basal levels of dissemination occurring in both subgroups with cells migrating either individually or collectively as clusters. Collective cerebellar tissue infiltration of SHH MB cells was further enhanced by EGF but not HGF, demonstrating differential tumour cell responses to microenvironmental cues. We found G3 cells to be hyper proliferative and observed aggressive tumour expansion even in the absence of exogenous growth factors. Our study thus provides unprecedented insights into brain tissue infiltration of SHH and G3 MB cells and reveals the cellular basis of the tumour progressing functions of EGF in SHH MB.

## Introduction

Medulloblastoma (MB) is the most common malignant paediatric brain tumour. It accounts for 15–20% of all childhood nervous system tumours^1^ with peak incidence rates at 6 children per million under 9 years of age^2^. Earlier thought of as a single disease entity, it is now well known that MB is comprised of at least four distinct molecular subtypes, namely, Wnt, sonic hedgehog (SHH), group 3 and group 4^3^. These subgroups remain stable in metastatic and recurrent disease^4^. MB arises in the cerebellum and is characterised by leptomeningeal dissemination to the brain and spinal cord. Surgery, radiotherapy and chemotherapy remain the current treatment modalities that have brought MB to a manageable condition for up to 70% of patients. However, MB survivors suffer from long lasting radiotherapy-induced side effects and despite the increasing understanding of the genetic and epigenetic hallmarks and differences in the different MB subgroups, an efficacious targeted therapy is still not available.

*In vitro* techniques used to address the lack of suitable therapy approaches do yield rapid results, which however may fall short of clinical relevance due to the failure of *in vitro* systems to mimic the complex natural tumour microenvironment. This is especially true in the case of long-established cell-lines that get easily adapted to the *in vitro* cell culture conditions. Therefore, genetically engineered mouse models of MB have been established to further study the molecular and cellular basis of tumour development. These include SHH signalling based models^5–7^, Tp53 mutation based models^8^, Sleeping beauty mutagenesis models^9,10^ and the MYCN-driven GTML mouse model^11^. On the one hand, these genetic mouse models allow straightforward evaluation of tumour development and monitoring of tumour size and location of the metastases. On the other hand, addressing local infiltration into the cerebellar tissue adjacent the tumour poses a challenge as the optimal time-point for analysis may greatly differ between the animals. An excellent alternative to genetic mouse models is the orthotopic implantation of patient-derived tumour cells and their further *in vivo* propagation^12–16^. However, accurate orthotopic implantation is a technically challenging approach and screening of therapeutic targets and testing the efficacy of potential drugs is rather inefficient and very costly using these models.

This calls for the development of an appropriate system that would use a normal brain component such as the cerebellum along with the MB tumour and bridge the current gap between *in vitro* and *in vivo* research. One such system is the organotypic cerebellar slice culture (OCSC), which entails the culturing, maintenance and long term survival of cerebellar slices *ex vivo* under physiological conditions^17^. This *ex vivo* model retains the cytoarchitecture as seen in the original tissue, and the extracellular matrix components closely resemble the *in vivo* situation. OCSCs have been widely used in neurobiology and brain slice cultures have recently also been used in the context of MB to test for the uptake and mobility of poly glycerol-adipate nanoparticles^18^ and for drug therapy using Smoothened antagonist LDE225^19^.

Marked genetic divergence in primary tumour compared to matched metastases have been described recently in experimental animal models and human patient samples^20^. This genetic divergence underscores the bicompartmental nature of primary and metastatic MB already recognized some time ago, when a set of putative metastasis driver genes had been identified^10^. Current models have thus focused on genetic events associated with or accumulated in metastases. Still largely unresolved questions are which of the metastasis-associated genetic events encode the cellular functions that drive dissemination away from the primary tumour and whether specific cellular or topological characteristics of the cerebellar microenvironment facilitate tissue infiltration. This is particularly relevant in light of the consensus reached recently on the high risk associated with metastatic MB, in particular also for SHH and group 3 MB^21^.

To identify intrinsic and microenvironmental mediators of brain tissue infiltration in MB, we have developed a cerebellar-MB tumour cell *ex vivo* co-culture system where SHH and Group 3 tumour spheroids are implanted on the organotypic cerebellar slice cultures. Using various molecular markers to identify the cellular components of the cerebellum by immunofluorescence and combining this with confocal microscopy, we have studied the dissemination and local infiltration of MB tumour cells. We demonstrate the suitability of this model for the efficient pre-clinical evaluation of anti-infiltration strategies, which would be instrumental to design and test novel treatment approaches as anti-metastatic therapies.

## Results

### The cerebellar slice- tumour cells co-culture

**Figure 1:**
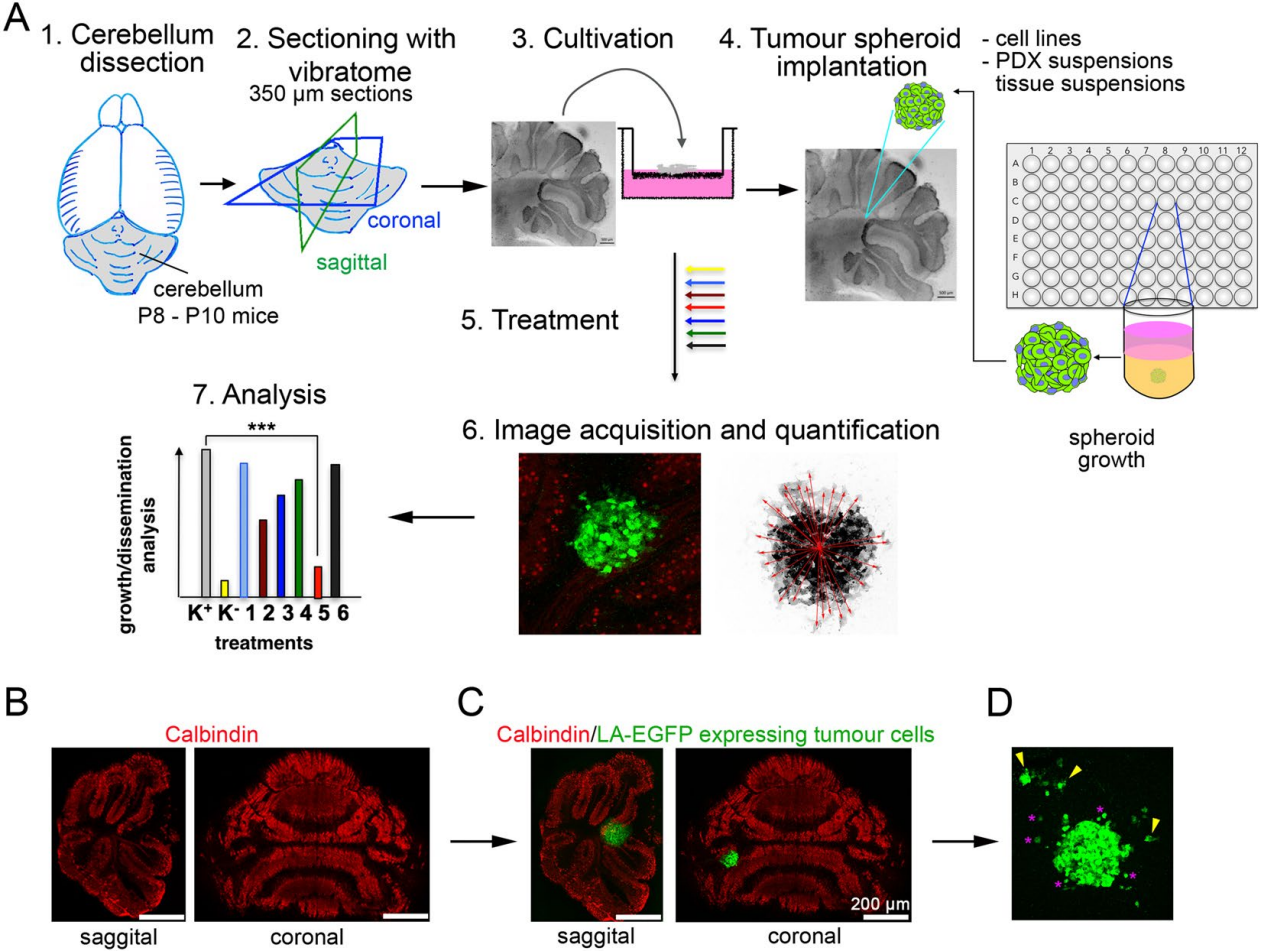
The organotypic cerebellar slice–tumour co-culture. **(A)** Workflow for OCSC generation and tumour spheroid implantation. (1) Decapitation of mouse pup(s) at PND 8–10 and isolation of cerebellum. (2) Sectioning of cerebellum under physiological conditions using a vibratome to generate 350 µm thick sagittal or coronal sections. (3) Culture of cerebellum sections on membrane inserts for 15 days *in vitro* (DIV). (4) Implantation of one LifeAct enhanced GFP (LA-EGFP) expressing tumour spheroid per slice. (5) Further incubation of the co-culture *in vitro* without or with treatment. (6) Fixation and immunofluorescence for microscopy analysis. (7) Quantification and data analysis. (**B)** Sagittal and coronal sections of cerebella, stained with anti-Calbindin (red) to visualise Purkinje cells. (**C)** Implantation of LA-EGFP expressing DAOY cells spheroid on the slices. (**D)** Dissemination of tumour cells (green) in the cerebellar slices after 5 days post initiation of culture.

In order to set up the *ex vivo* model, cerebella were dissected from mice pups at postnatal day (PND) 8–10, sliced and put in culture under physiological conditions (Fig. 1A). PND 8–10 corresponds approximately to the neurodevelopmental stage of a new-born infant^22^. Since one of the locations for the occurrence of childhood MB is close to the vermis, cerebella were oriented in such a way that during sectioning we either obtained typical lobulated sagittal slices or coronal sections containing the vermis (Fig. 1B). The 350 µm thick slices were cultivated on membrane inserts (placed in a six well plate containing medium) for a suitable period of time. Spheroids of DAOY MB cells expressing LifeAct enhanced GFP (LA-EGFP) were then implanted on the cerebellar slices (Fig. 1A, C). One spheroid was implanted per slice and this was verified under the microscope one day post spheroid implantation. This organotypic cerebellar slice-tumour spheroid co-culture system was further maintained *in vitro* and the study of growth and infiltration of tumour cells was carried out using immunofluorescence and confocal microscopy. We observed that there was a basal level of dissemination in the slices where tumour cells were migrating either as single cells (asterisk) or in clusters (arrowheads) (Fig. 1D).

### Determination of a suitable time point for the implantation of tumour spheroids and study of tumour cell dynamics in the OCSCs

**Figure 2:**
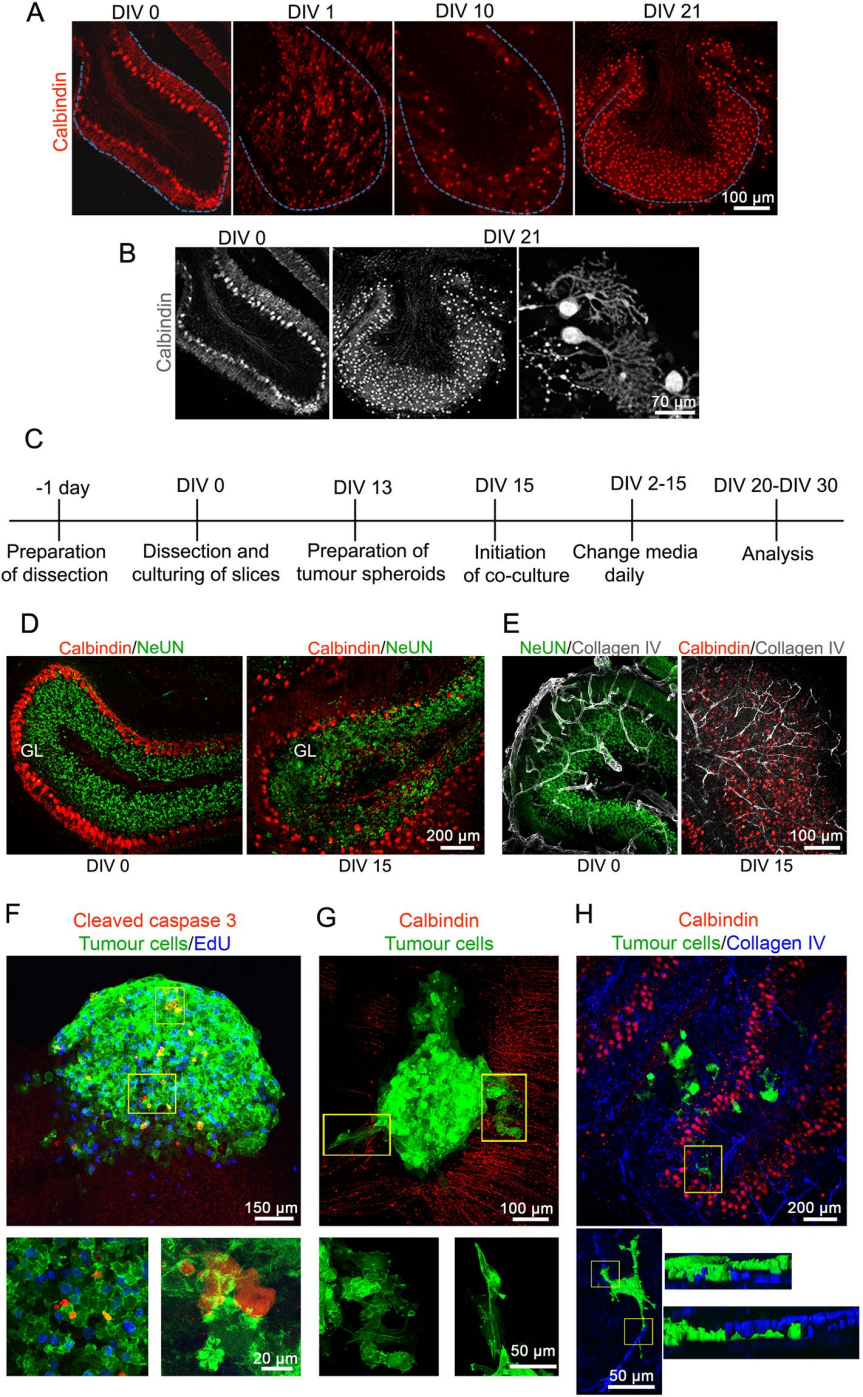
Morphological assessments of the cerebellar slices and co-culture *in vitro*. (**A)** Changes in the Purkinje cell monolayer upon *in vitro* culturing of the slices was monitored at DIV 0, DIV 1, DIV 10 and DIV 21 with anti-calbindin antibody (red) using confocal microscopy. **(B)** Grey scale images of cerebellar slices at DIV 0 and after being cultured for DIV 21. (**C)** Timeline of the experimental setup for five-day co-culture experiment. DIV 15 was selected as the time point suitable for the implantation of tumour spheroid. (**D)** The cerebellar cortex layers visualised using confocal microscopy at DIV 0 and DIV 15 for comparison with anti-Calbindin (red, Purkinje cell layer) and anti-NeuN (green, granular layer) antibodies. (**E)** Visualisation of the vasculature using anti-collagen IV (grey) at DIV 0 and DIV 15. (**F)** Determination of apoptosis and proliferation after five-day co-culture using anti-cleaved caspase 3 (red) and Click-iT EdU (blue), respectively. (**G)** Monitoring the tumour cells infiltrating the cerebellar slices. (**H)** Visualization of the vasculature (blue) in the presence of tumour cells. Lower panels: zoomed versions of boxed insets in F, G and H respectively. In H, EGFP and Collagen IV fluorescence was volumised.

A critical step in the establishment of the co-culture model is determining the time point suitable for the implantation of the tumour spheroid. Hence, we studied the morphological changes in the cerebellar architecture of the slices at various time points namely: day *in vitro* (DIV) 0, DIV 1, DIV 10 and DIV 21 using immunofluorescence. We observed that at DIV 1, the characteristic Purkinje cell monolayer (red) observed at DIV 0 was lost. This could be due to the inevitable trimming of certain axons during cerebellar sectioning. However, as the culturing time progressed, a gradual re-organisation in the slices was observed, possibly due to the establishment of new synaptic connections. Between DIV 15 and DIV 21, an orderly arrangement of the Purkinje cells was visible in most parts of the culture. By DIV 21, the thickness of the slices was reduced and the Purkinje cell layer (PCL) was established in close proximity to the arrangement at DIV 0 (Fig. 2A). Furthermore, between DIV 10 and DIV 21, the presence of axon collaterals that ran into the inner granular layer and dendritic spines that formed extensive branches indicated slice maturation (Fig. 2B). Based on this, DIV 15 was selected as a time point suitable for the implantation of the tumour spheroids and the slice culture protocol was accordingly adjusted. Figure 2C shows the timeline of the procedures with the analysis of the implanted spheroids between 5 and 15 days after implantation (DIV 20 – DIV 30). Using markers to identify the different layers within the cerebellar cortex (Fig. 2D) and the vasculature (Fig. 2E), we investigated any possible alterations in tissue organization between DIV 0 and DIV 15. At DIV 15, we observed a clearly layered organization in lobules with the granular layer (GL) embedded within an array of Purkinje cells. The robust vasculature observed using anti-Collagen IV staining at DIV 0 appeared to thin down/flatten and reorganise with new endothelial network in mature slices at DIV 15. To evaluate the extents of proliferation both in the tumour cells as well as the slice culture at DIV 20 (5 days of co-culture), we performed 5-ethynyl-2′-deoxyuridine (EdU) staining followed by detection with a fluorescent azide through a Cu(I)-catalysed [3 + 2] cycloaddition reaction (“click” chemistry). To determine the number of apoptotic cells, we stained for cleaved caspase 3. We found that the LA-EGFP-positive tumour cells displayed a mixed population of cells – with a majority of cells proliferating and less than 10% of them positive for cleaved caspase 3 (Fig. 2F). A few EdU-positive cells were also detected in the slice, while caspase 3 cleavage levels above background were not detectable. When the co-culture was maintained for 5 days *in vitro*, the disseminating tumour cells showed characteristic lamellipodia and mesenchymal morphology with a rear-front cell polarity (Fig. 2G). Upon 15 days of co-culture initiation, we observed distinct migration of tumour cells and these migrating cells were in the vicinity of the endothelial network (Fig. 2H).

We concluded that the optimal time-point for tumour spheroid implantation is at DIV 15. Implanted tumour cells proliferate and only a small proportion of the tumour cells show signs of cell death. Thus, the co-cultured tumour cells are viable, proliferative, infiltrate the cerebellar tissue and do not affect the viability of the cellular components of the host tissue.

### Qualitative and quantitative study of MB cell migration and tumour mass using OCSC

**Figure 3:**
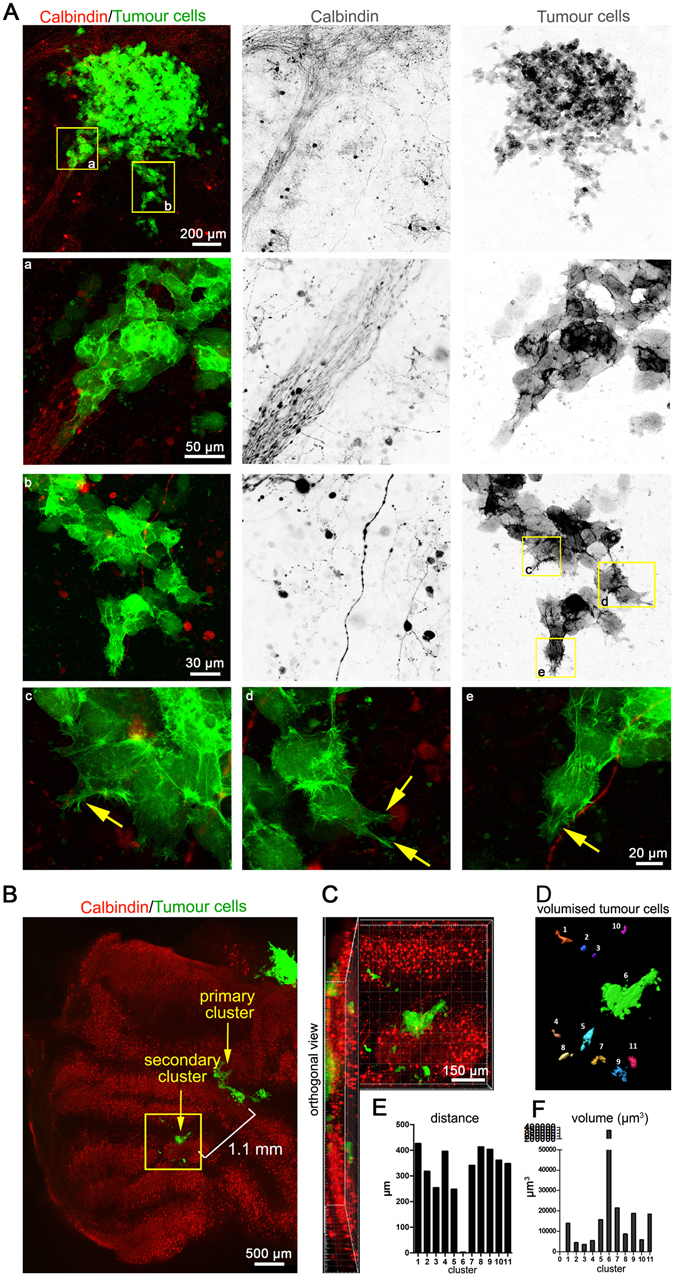
Qualitative and quantitative analysis of MB cell behaviour in co-culture. **(A)** Visualisation of the co-culture initiated for 5 days. Morphological assessment of the invading cells was performed (boxed insets a to e and their respective zoomed in versions). **(B)** Purkinje cell/tumour cell co-culture visualisation 12 days after implantation. (**C)** Zoomed version of the boxed area in A and its orthogonal view. (**D)** Volumisation of the tumour clusters in B using Imaris. Graphical representation of the distance of the satellite tumour cells from the tumour cluster 6 **(E)** and tumour volume **(F)**. Inverted grey scale images show individual channels for better visualisation of cellular structures. a - e are 63x zoomed-in images of the yellow boxed areas. Arrows point to invasive lamellipodia- and filopodia-like protrusions in invading cells.

Qualitative and quantitative analysis of MB cell dissemination are essential prerequisites for the evaluation of tumour cell behaviour, especially after the application of an experimental preclinical treatment. Since the infiltration of the healthy cerebellar tissue by transformed MB cells requires cell motility, we qualitatively assessed the morphological characteristics of migrating MB cells in the cerebellar slices after 5 days of co-culture initiation. We used DAOY MB cells expressing LA-EGFP to allow visualisation and analysis of the cytoskeleton. We observed that even in the absence of an exogenous stimulation, MB cells infiltrated in a mixed phenotype with both single and clusters of infiltrating cells (Fig. 3A). Infiltrating cells were elongated and characterised by long extensions and marked lamellipodia and filopodia-like protrusions comprised of F-actin mesh works and bundles (Fig. 3A, c-e, arrows). We also observed brain tissue infiltration with the human SHH MB line UW228 (Fig. S2), the G3 MB line HD-MB03 (Fig. 5B), as well as the two primary glioblastoma cell lines ZH411 and ZH561 (Fig. S1).

For quantitative analysis, we allowed the tumour cells to grow and disseminate for 12 days after implantation into a coronal section. The co-culture was then fixed and stained for calbindin and microscopically investigated (Fig. 3B). We observed distinct separation of a secondary cluster of tumour cells from the primary tumour cell cluster and active further dissemination of LA-EGFP-positive cells inside the cerebellar tissue. Orthogonal sections revealed that these cells had indeed infiltrated into the tissue and were not present just at the surface (Fig. 3C). To determine how far the cells had disseminated from the secondary cluster in the tissue, we quantified the distance of migration of the tertiary clusters from the centre of cluster 6 (secondary cluster, Fig. 3D). We volumised the individual secondary and tertiary clusters (Fig. 3C and D) and quantified their volumes to get insight in their relative sizes. We quantified volumes around 5000 to 10,000 μm^3^ in the tertiary clusters (Fig. 3F) and localized them in a distance of 200 to 400 µm from secondary cluster 6 (Fig. 3E).

These data show that OCSCs can be used to study the migration dynamics of MB cells in the cerebellar slices and to quantitatively address dissemination distances and volumes of individual tumour cells/clusters. They furthermore indicate that the disseminating cells either individualise immediately after cell division or do not proliferate massively and remain quiescent.

### EGF promotes sheet-like cerebellar tissue invasion in cell model of SHH MB

**Figure 4:**
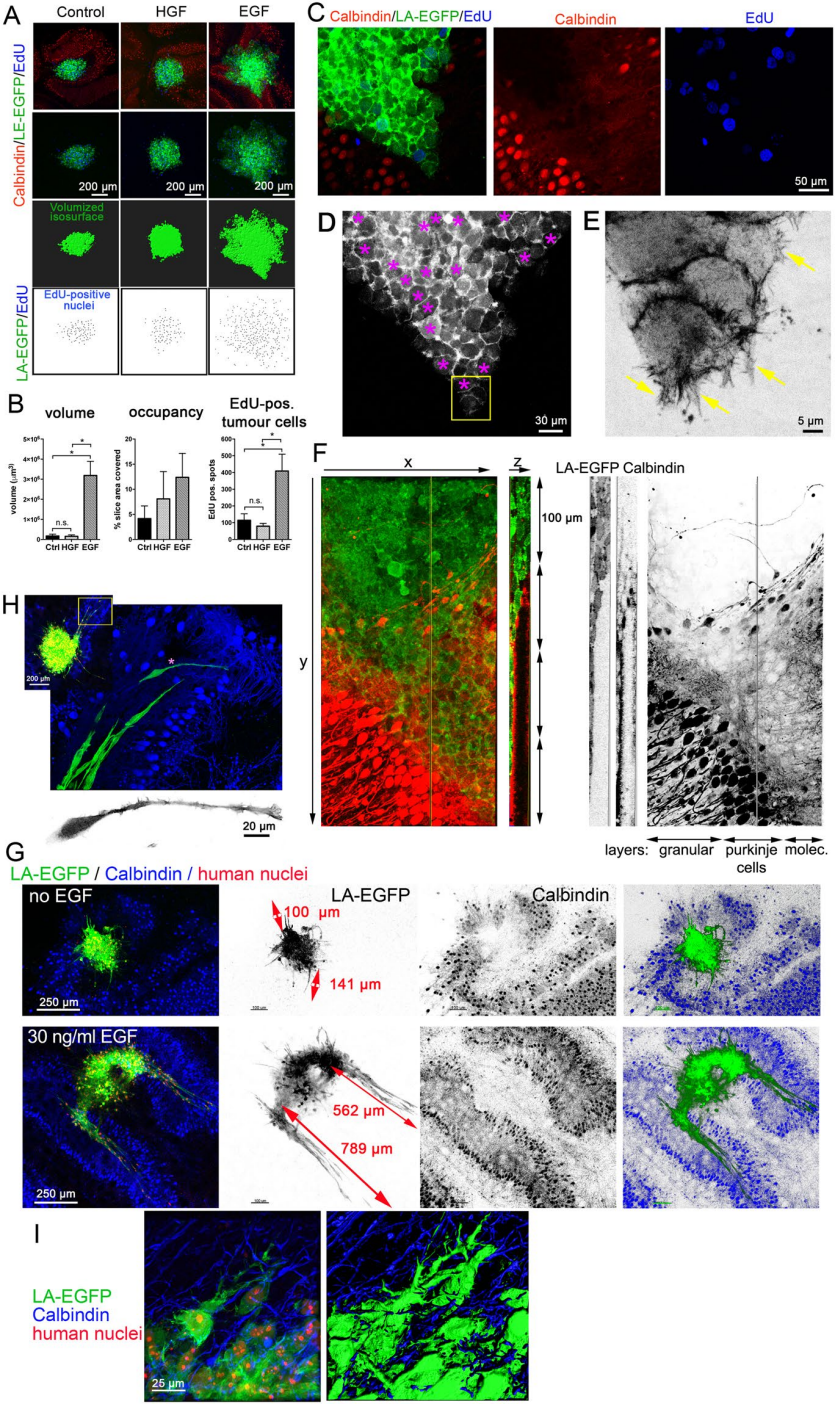
Quantification of relative growth and proliferation in response to HGF or EGF stimulation. **(A)** Confocal microscopy analysis of DAOY LA-EGFP cells spheroid co-cultured for five days in the OCSCs and exposed to the growth factors as indicated. EGFP signal was volumised and volume quantified. EdU-positive nuclei were marked and counted as well. (**B)** Quantification of volume, occupancy (tumour area/slice area) and number of EdU-positive nuclei. Mean and SD of two independent experiments are shown. ANOVA statistical analysis was performed (* = p < 0.05). (**C)** Higher magnification of EGF-stimulated DAOY cells invading the molecular layer. **D)** Purple asterisks in LA-EGFP grey scale panel highlight the proliferating cells. **E)** Magnification of the invasion front (boxed inset in (**D**). Inverted grey scale image shows F-actin cytoskeleton of leading edge cells with distinct filopodia and lamellipodia (arrows). (**F)** EGF-induced invasion of DAOY cells into molecular layer (green: LA-EGFP, red: anti-Calbindin). Orthogonal XYZ section shows infiltration of tumour cells into Purkinje cell layer. Red and green channels are also shown as inverted grey scale images for better visualization of the different layers. (**G)** Comparison of growth and invasion of untreated and EGF-stimulated UW228 tumour spheroids. Red arrows in LA-EGF grey-scale image indicate approximate length of invading cell streams. (**H)** Higher magnification of tissue infiltration of UW228 cells in response to EGF. Leader cells (green) at invasion front 600 µm deep in the host tissue and Purkinje cells (blue) are shown. Inverted grey scale image shows elongated F-actin cytoskeleton of the leader cell with pronounced lamellipodium. (**I)** Morphology of EGF-stimulated UW228 cells during spheroid-proximal infiltration. Image to the right shows volumised F-actin cytoskeleton with marked invasive protrusions.

In previous studies, we identified hepatocyte growth factor (HGF) and epidermal growth factor (EGF) as powerful pro-migratory growth factors for MB *in vitro*^23,24^. In order to determine their biological relevance for MB tissue infiltration, we treated cerebellar slice-MB tumour cell co-culture with either HGF or EGF and measured the change(s) in the tumour cell dynamics/tumour cell growth and proliferation compared to the untreated control after five days of continuous treatment. Interestingly and in contrast to the data of our *in vitro* studies, HGF did not stimulate migration over and above what we observed in untreated cells. In contrast, EGF elicited a very distinct tumour cell behaviour, both in DAOY (Fig. 4A) and UW228 (Fig. 4G) cells. In parallel, we also determined the number of nuclei having incorporated EdU, to estimate differences in proliferation under the three conditions. A much larger number of EdU-positive nuclei were detected in EGF-stimulated cells compared to HGF-treated or control cells (Fig. 4B). Tumour cells under EGF treatment also occupied a markedly larger slice area than in untreated slices (Fig. 4B). Consistently, the quantification of the volume of the LA-EGFP signal in the image stacks acquired from EGF-treated slices displayed a significant increase in the tumour volume compared to the untreated control (Fig. 4B).

Along with this, a remarkable difference in the mode of growth and infiltration/spread was observed under EGF-treatment, where DAOY cells expanded into the cerebellar tissue in a compact sheet in which cell-cell contacts remained intact. The invasion front of the EGF-stimulated cells was oriented towards the molecular layer (Fig. 4C). EdU incorporation indicated no difference in proliferation between the invading and non-invading cells as EdU positivity could be seen both at the front of invasion as well as further inside (Fig. 4D, asterisks). The invading cell sheets observed in response to EGF stimulation appeared to be guided by a few leader cells at the invasion front. Remarkably, these leader cells displayed pronounced lamellipodia, which extended F-actin-rich, filopodia-like protrusions into the cerebellar tissue. (Fig. 4E, arrows). Similarly, UW228 cells invade collectively when stimulated with EGF (Fig. 4G). However, unlike DAOY cells, UW228 tend to form streams of elongated cells that extend several hundred microns deep into the molecular layer of the surrounding brain tissue (Fig. 4G,H). Marked, F-actin polymerization-driven protrusions are also clearly detectable in cells invading in close proximity of the tumour spheroid (Fig. 4I).

Together, these experiments confirmed EGF as a strong promoter of cell proliferation and migration in MB and demonstrated its potential to drive brain tissue infiltration of the tumour cells. This demonstrates that the *ex vivo* model can be used as a tool for testing the potential of various growth factors and screening of probable therapy targets. Our data furthermore show that during EGF-driven tissue invasion, the infiltrating cell mass is organized in sheets or as streams with leader cells extending marked, F-actin-rich protrusions at the invasion front that penetrate preferentially into the molecular layer.

### SHH and group 3 MB cell grow differently in brain tissue

**Figure 5:**
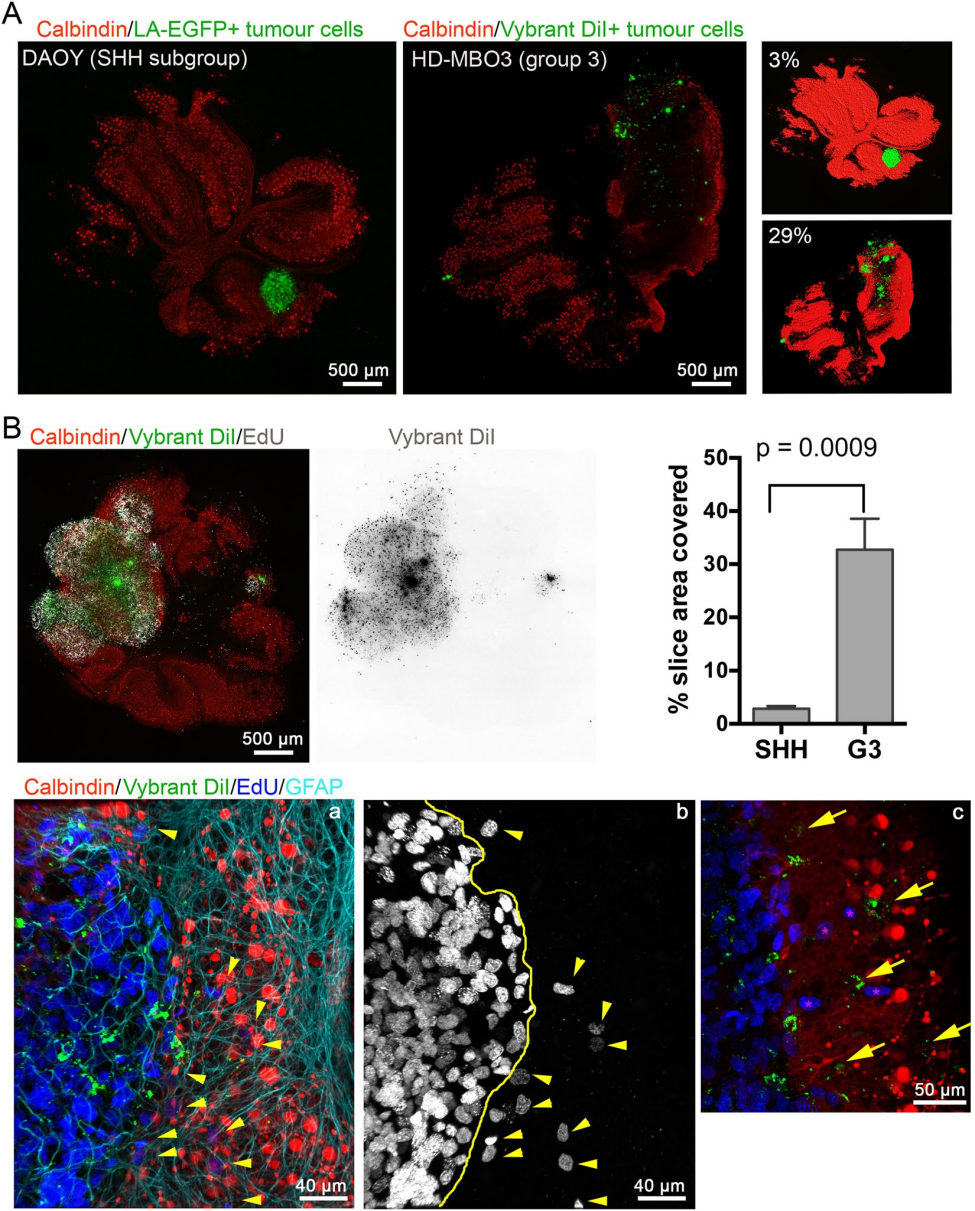
Massive growth and brain tissue infiltration of group 3 MB cells. (**A)** LA-EGFP DAOY or HD-MBO3 cell spheroids were implanted in DIV 15 cerebellar slices and incubated for 5 days. Image show confocal microscopy image analysis of whole slices. Staining as indicated. Tumour area relative to whole cerebellar slice area was quantified (right panels) and was 2.9 and 29.0% for DAOY and HD-MBO3, respectively. (**B)** Growth of HD-MBO3 spheroid in OCSC and microscopy analysis as in (A). Inverted grey scale images show Vybrant Dil staining of the tumour cells. Graphical representation of the occupancy obtained from three independent experiments for each cell line. Means and SD are shown with statistical analysis done using unpaired, two-tailed t-test. (a) is a higher magnification confocal image of the tumour-parenchyma border. Yellow line in (b) indicates tumour border and yellow arrowheads EdU-positive nuclei of infiltrating tumour cells. (c) as (a) but with yellow arrows pointing to EdU-negative infiltrating tumour cells identified by Vybrant Dil staining (green). Asterisks indicate EdU-positive nuclei of infiltrating tumour cells.

Our studies using DAOY and UW228 cells have revealed novel morphological characteristics of growth and infiltration of these SHH MB lines. To extend this study and to identify differences in growth and infiltration behaviour between cells of different molecular subgroups, we set up OCSCs with group 3 MB cells to establish the novel *ex vivo* model also for group 3 MB. We chose this subgroup because metastasizing group 3 MB is very difficult to manage in the clinic and associated with very high risk and poor prognosis^21^. We used HD-MBO3 cells that were isolated from a metastasizing group 3 MB and which can be grown both *in vitro* and *in vivo*^25^. Similar to the SHH MB DAOY cells, HD-MB03 cells were grown into spheroids and then implanted in the cerebellum slices according to the protocol established for DAOY spheroids. As HD-MBO3 cells expressing LA-EGFP are not yet available, we visualised the tumour cells using either EdU staining or Vybrant Dil or both (Fig. 5A). 5 days after implantation of the spheroid, we detected a very large tumour cell mass that had expanded within the cerebellum. We compared the relative area covered by the SHH and group 3 tumour cell masses in the cerebellar slices. The HD-MBO3 cells expanded nearly three-fold more than the DAOY cells and covered 29% of the slice area (SHH: 3%) (Fig. 5A, right, 5B). Using another HD-MBO3-cerebellar co-culture slice, we determined whether group 3 MB cells also infiltrate the cerebellar tissue (Fig. 5B). We zoomed into the tumour border region and detected EdU-positive nuclei of the same size and appearance like those detected in the main tumour mass (Fig. 5B a & b, arrowheads). Since not all HD-MBO3 cells are expected to incorporate EdU, we labelled the tumour cells with the lipophilic membrane stain Vybrant Dil prior to implantation, which should allow visualisation of the tumour cells by fluorescent microscopy. However, due to the very high proliferation rate, the dye diluted out and was detectable as perinuclear spots and not, as expected, in the plasma membrane (Fig. 5B). Nevertheless, the Vybrant Dil detection also identified EdU-negative tumour cells that had infiltrated into the brain tissue (Fig. 5B c, arrows c), thus confirming that HD-MBO3 expansion in the brain slice also involves tissue infiltration of solitary tumour cells.

Thus, OCSC is also suitable for growing group 3 MB cells. Importantly, the growth rate of group 3 cells is markedly higher that of the SHH MB lines DAOY or UW228. Single HD-MBO3 tumour cells inside the cerebellar tissue can be detected using the proliferation marker EdU or the lipophilic stain Vybrant Dil, suggesting that HD-MBO3 expands by both growth and local infiltration.

## Discussion

To overcome the lack of appropriate methods for the investigation of local tissue infiltration in metastatic MB, we successfully established an organotypic cerebellar slice-MB tumour co-culture model system. We demonstrated that the host cerebellum tissue and the co-cultured tumour cells survive for up to 30 days *ex vivo*. Furthermore, the tumour cells propagated, suggesting that there was no rejection of the tumour spheroids (human) by the host tissue (mouse). We found that the cerebellar cytoarchitecture was well preserved and detected infiltrating tumour cells distant from the primary tumour spheroid. Using standard imaging software, we showed that short-range dissemination of tumour cells and tumour volume can be quantified. Furthermore, the treatment of MB cells of the SHH subtype with the growth factors HGF or EGF revealed a factor-specific response, wherein EGF strongly promoted growth and collective infiltration of the brain tissue. In contrast, HGF treatment induced single cell dissemination similar to the untreated control and did not cause an increase in tumour volume. The comparison of MB cells of the molecular subgroup SHH and group 3 revealed a clear growth advantage of latter. In conclusion, this novel *ex vivo* model is a valuable and versatile tool for basic and translational MB research. It mimics the initial stages of cerebellar tissue infiltration, and it can thus be used for the identification of growth and dissemination promoting molecular pathways and for evaluating targeting strategies to block them.

One important aspect is the implantation of MB tumour spheroids into cerebellar slices of neonatal mice, as opposed to the distribution of MB tumour cells on whole brain slices^19^ or to the implantation of tumour cells into a hole punctured into sliced human brain tissue^26^. We consider the OCSC and spheroid implantation approach favourable because (i) tumour cells grown as spheroids can recapitulate the natural cell-matrix interactions, and have gene and protein expression profiles closer to the parental tumours compared to cells grown as a monolayer^26–29^, (ii) the time point used for the dissection of the cerebellar slices in this study corresponds to the developmental stage of infants, one of the target populations in MB and iii) the use of spheroids facilitates to observe and calculate the range of dissemination from the site of implantation. Latter is especially helpful when investigating the effects of compounds targeting cell migration and invasion. Previous studies^19,30^ used whole brain slices of P6 mouse pups cultivated for 24 to 48 hours before the addition of tumour cells. This approach poses a marked risk of reactive gliosis that may affect tumour cell behaviour in an unpredictable manner. Moreover, its is well known that cerebellar brain slices reorganize during maturation^31^ and our analysis of the purkinje cell re-distribution, which is completed around DIV15, strongly argues for choosing a time point of DIV 15 or more for implantation.

The treatment of co-cultured SHH MB cell spheroids with EGF for five days caused massive and well quantifiable dissemination of the tumour cells. This EGF-induced dissemination of MB cells occurs mostly in the molecular layer, suggesting that cell density and composition may guide spreading of MB in the brain. In contrast, when the co-culture was treated with HGF alone, no appreciable increase of tumour mass over the controls was observed using volumetric analysis despite a moderate increase in slice occupancy. This data diverged from the previously described pro-migratory function of HGF in MB^23,24,32,33^. Although we do not fully understand this discrepancy yet, it is possible that HGF stimulation causes a diffuse infiltration of single cells into the cerebellum tissue with no apparent proliferation of the primary tumour mass. A similar observation with diffuse brain slice infiltration and a reduction of the spheroid mass was noted with primary glioblastoma cells^34^ and our study using primary glioblastoma-derived cells confirmed diffuse infiltration with small increases in volume and cell number only. Upon extended co-culture (12 + days), we observed that the single infiltrating cells displayed reduced LA-EGFP expression. We tried to highlight EGFP-dim cells using anti-CD47 (data not shown), which clearly labelled the tumour cells in the spheroid but failed to clearly detect the disseminated cells. Thus, there is a possibility wherein single MB tumour cells in untreated/HGF co-culture did disseminate effectively as well but their microscopy detection failed due to low fluorescence. Hence, for the analysis of diffusely infiltrating cells, the establishment of a fluorophore tagging strategy ensuring bright fluorescence is an essential prerequisite that needs to be established in a cell and condition-specific manner. As an alternative approach, we recommend using anti-human nuclei antibody, which highlights the nuclei of the human tumour cells only and can therefore be easily used to detect primary human tumour material in short- and long-term co-culture experiments.

The formation of F-actin-rich, lamellipodia and filopodia-like protrusions at the leading edge of invading cells hallmark both single and collective cell infiltration. It is thus conceivable that those protrusions are not only associated with but causative for cerebellar tissue infiltration, as was recently proposed more generally for the invasion of solid tumours^35^. During collective invasion after EGF stimulation, filopodia-like protrusions are apparent predominately in the leader cells that head the infiltrating cohort. How these protrusions of the MB cells are regulated in the brain tissue is not known but a better mechanistic understanding of their regulation may provide clues for targeting invasion and dissemination. Indeed, we previously implicated Ser/Thr kinase MAP4K4 in lamellipodia formation in MB cells invading collagen gels in response to HGF stimulation^23^. It now remains to be determined whether MAP4K4 also controls protrusion formation during tissue invasion.

We found the group 3 HD-MBO3 MB line to grow aggressively in the cerebellar slices with an expansion rate nearly three times higher than that of the SHH DAOY line. Although enhanced proliferation rates of this line probably account for most of the increased growth, it will be of outmost interest to determine whether the infiltration of single cells we detect or of whole cell clusters may also be a contributing factor. Therefore, future studies of HD-MBO3 cells stably expressing appropriate tags for microscopic visualisation combined with migration/invasion inhibition will be necessary to clarify the contribution of cell dissemination capability to tumour expansion, as was proposed recently in a seminal publication^36^.

Mutual interactions between the tumour cells and the host tissue have a major influence on the promotion of tumour development, tumour progression as well as chemo and radiosensitivity^37^. These interactions can now be mimicked in OCSCs and they will enable the identification of factors that support MB tumour growth and eventually, also resistance to therapy. In this way, the co-culture system surpasses *in vitro* tools which are limited to using specific extracellular matrix components such as collagen I or matrigel, which neither mimic the morphological nor the chemical complexity of the cerebellar parenchyma. Along the same lines, we have also detected the infiltrating tumour cells in the vicinity of or in contact with the blood vasculature. Despite the lack of vascular supply to the slices, we and others^38^ found that the capillary endothelial cells do survive in these sections and reorganise into tube like structures. Thus, the capillary endothelial cells most likely remain capable of expressing and secreting various molecules, which could influence other cells in the slice culture including the tumour cells. However, unlike breast and lung carcinoma cells metastasizing to the brain^39^, MB cells do not colonize the vascular network in the brain slices and the potential significance of their rather loose interaction with the endothelial network remains to be determined.

Together, we have established and validated OCSCs as a versatile platform for preclinical research of paediatric brain tumours. It will greatly facilitate the search for novel therapy strategies targeting growth and dissemination for MB and other primary brain tumours. In addition to the great spatio-temporal insight OCSCs offer in growth and dissemination behaviour of brain tumour cells, they also help to reduce the number of animals used in pre-clinical research while increasing the screening capabilities for drug testing studies.

## Methods

### Cells and cell lines

DAOY human MB cells were purchased from American Type Culture Collection (ATCC, Rockville, MD, USA) and were transduced with lentivirus pLenti-LA-EGFP^23^. UW228^40^ was generously provided by John Silber (Seattle, USA). DAOY and UW228 cells were cultured as described in^41^. The human glioblastoma primary cell lines ZH411 and ZH561 were generously provided by the Department of Neurology of the University Hospital Zürich. ZH411 and ZH561 were originally isolated from surgically removed *de novo* glioblastomas and cultured in neurobasal medium supplemented with 2% B27, 1% glutamine, epidermal growth factor (EGF, 10 ng/ml), and basic fibroblast growth factor (bFGF, 20 ng/ml)^42^. HD-MBO3 group 3 MB cells were obtained from Till Milde and grown as described in^25^.

### Animals

Wild type C57BL/6JRj pregnant females were purchased from Janvier Labs and were kept in the animal facilities of the University of Zürich Laboratory Animal Centre. Mouse protocols for organotypic brain slice culture were approved by the Veterinary Office of the Canton Zürich.

### Reagents

Antibodies: goat anti-GFAP (ab53554, 1:300), rabbit anti-Calbindin (ab11426, 1:1000), mouse anti-Calbindin (ab82812, 1:1000), donkey anti-rabbit (ab175649, 1: 100) all from Abcam and donkey anti-goat (A11057, 1:500), Vybrant CM-DiI cell labelling solution (V-2288) from Life Technologies, mouse anti-NeuN clone A60 (MAB377, 1 in 100), mouse anti-GFAP (MAB360, 1:500), rabbit anti-collagen type IV (AB756P, 1:500), anti-nuclei clone 3E1.3(MAB4383) from Millipore, rabbit anti-cleaved caspase 3 (5A1E, 1:500) from Cell Signaling Technology, Click-iT EdU Alexa Fluor-647 imaging kit from Molecular Probes. HGF and EGF were ordered from Peprotech and used at a final concentration of 50 ng/mL and 30 ng/mL respectively. All the OCSC culture reagents have been described in^43^.

### Organotypic cerebellar slice culture (OCSC)

The experimental set up followed and reagents used were similar to the one described in^43^. Briefly, wild type C57BL/6JRj mice pups were sacrificed at postnatal day (PND) 8–10 by decapitation. Cerebella were dissected and placed in cold Geys balanced salt solution containing kynurenic acid (GBSSK). These were then embedded in 2% low melting point agarose gel in an orientation dependent on whether coronal or sagittal sections were needed. After the gel had solidified, the agarose block was glued onto the vibratome (VT 1200 S, Leica) disc with Roti Coll1 glue (0258.1 Carl Roth). The disc was then mounted in the vibratome chamber filled with cold GBSSK and 350 μm thick sections were cut. After removal of excess agarose around the slices, they were transferred to petri dishes filled with cold GBSSK. Millipore inserts (PICM 03050, Merck Millipore) were placed in six well plate(s) filled with 1 mL cold slice culture medium (SCM) onto which the slices were then transferred using a Rotilabo-embryo spoon (TL85.1, Carl Roth). A maximum of three slices were placed per insert and excess of medium was removed. Slices were monitored for any signs of apoptosis and media was changed daily for the first week and once in two days thereafter.

### Formation of tumour spheroids and initiation of co-culture

For spheroid formation, 2500 cells/well were seeded in a 96 well Lipidure-Coat plate A-U96 (AMS-51011610, Amsbio). and incubated for 48 hours at 37 °C, 5% CO_2_, 95% humidity to form spheroids. For spheroids from DAOY and UW228 cells, medium contained: Improved MEM (Gibco, 10373-017), 10% FBS and Pen/Strep solution, while for spheroids from HD-MBO3 cells, medium contained: DMEM (Sigma, D5761), 10% heat inactivated FBS, L- glutamine and Pen/Strep solution. Post incubation, the spheroids, of a diameter of approximately 150–200 µm, were collected from each well into a Falcon tube and were left for 5 minutes at room temperature (RT). Once all the spheroids had sedimented to the bottom of the tube, the medium was aspirated and the spheroids were re-suspended in 5 mL of SCM. 100 μl of suspension was collected ensuring it contained one tumour spheroid/slice and implanted by placing it on top of the slice at 15 days *in vitro* (DIV) after sectioning. Excess medium was aspirated out and the co-culture was maintained at 37 °C, 5% CO_2_, 95% humidity for at least 5 days.

### Immunofluorescence protocol

The inserts were placed in a six well plate containing 1 mL of cold 4% PFA made in Phosphate-buffered saline (PBS) at pH 7.4. Additionally, 1 mL of cold 4% PFA was also added on top of the insert(s) and incubated at RT for 1 hour on a shaker. After three washes, 10 mins each with 1X PBS, the part of the insert, which contained the slice(s) was carefully cut under a dissecting microscope using a scalpel. The slices were then incubated in standard cell culture trypsin EDTA (250 μl/well of a 24 well plate) and incubated at 37 °C humidified incubator for 23 minutes. The slices were then blocked in blocking solution (BS) containing 3% foetal calf serum, 3% bovine serum albumin and 0.3% triton x 100 for 1 hour at RT. Primary antibodies were diluted in the same BS and incubated overnight on a shaker at 4 °C. Following 3 washes at RT using 5% BSA in PBS, secondary antibodies were incubated for 3 hours at RT. The inserts were flat mounted in glycergel mounting medium (C0563, Dako). All images were acquired on a Leica SP8 inverted confocal microscope.

For assessing proliferation, Click-iT EdU Alexa 647 Imaging kit was used (C10340, Invitrogen). Exact protocol as described in the kit was followed. 10 μM EdU was used and the Click-iT reaction cocktail was incubated for 45 mins at RT. Immunofluorescence protocol was the same as above.

Vybrant Dil staining was performed as per manufacturer guidelines.

### Microscopy and analysis

Images were acquired using Leica TCS SP8 inverted microscope and processed/analysed using ImageJ. Quantification of volume and EdU-positive nuclei count was done using Imaris. Volume and area were quantified by rendering an isosurface. EdU positive nuclei were counted using the spots function and only those EdU positive spots under the GFP channel were selected.

### Ethical approval

All experiments were carried out in accordance with the guidelines and regulations of the University of Zürich. The *ex vivo* experiments using cerebella of ten days old mice was approved by the veterinary office of the Kanton of Zürich.

## Electronic supplementary material

Figure S1
