# Publisher Correction: Investigation of brain tissue infiltration by medulloblastoma cells in an *ex vivo* model

**DOI:** 10.1038/s41598-018-28051-3

**Published:** 2018-06-21

**Authors:** Anuja Neve, Karthiga Santhana Kumar, Dimitra Tripolitsioti, Michael A. Grotzer, Martin Baumgartner

**Affiliations:** 1 Children’s Research Centre, Oncology, University Children’s Hospital, August-Forel Strasse 1, CH-8008 Zürich, Switzerland; 2 Department of Oncology, University Children’s Hospital Zürich, Steinwiesstrasse 75, CH-8032 Zürich, Switzerland

Correction to: *Scientific Reports* 10.1038/s41598-017-05573-w, published online 13 July 2017

In the original version of this Article, the author Karthiga Santhana Kumar was incorrectly indexed. This error has now been corrected.

