# Supplementary material for: Investigation of brain tissue infiltration by medulloblastoma cells in an ex vivo model
Source: Sci Rep. 2017 Jul 13;7:5297. doi: 10.1038/s41598-017-05573-w (PMC5509741; doi:10.1038/s41598-017-05573-w)
Supplement: Supplementary file 1 — Figure S1 [file 41598_2017_5573_MOESM1_ESM.pdf]

Investigation of brain tissue infiltration by medulloblastoma cells in an *ex vivo* model

Anuja Neve[1], Karthiga Santhana Kumar[1], Dimitra Tripolitsioti[1], Michael Grotzer[1,2] and Martin Baumgartner[1,*]

[1] Children's Research Centre, Oncology, University Children's Hospital, August-Forel Strasse 1, CH-8008 Zürich, Switzerland

[2] Department of Oncology, University Children's Hospital Zürich, Steinwiesstrasse 75, CH-8032 Zürich, Switzerland

*Correspondence to: Martin Baumgartner, University Children's Hospital Zürich, Children's Research Centre, Dept. of Oncology, Paediatric Neuro-Oncology group, August-Forel Strasse 1, CH-8008 Zürich,
E-mail: Martin.Baumgartner@kispi.uzh.ch, Phone: +41 44 634 88 20, FAX: +41 44 634 88 59

Keywords:

Organotypic cerebellar slice culture, medulloblastoma, epidermal growth factor, cell motility and invasiveness/infiltration dynamics, molecular subgroups

Fig. S1

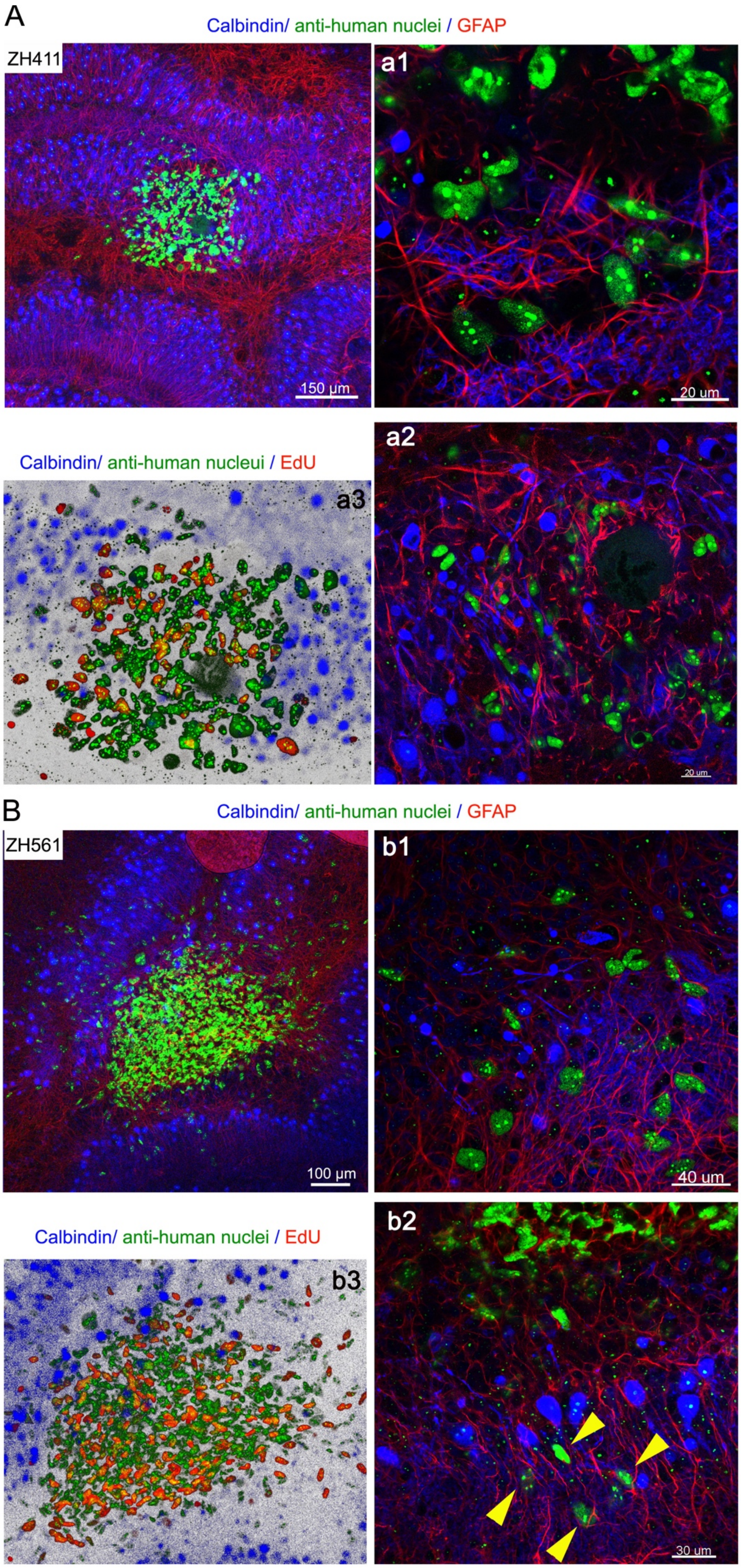
A
Calbindin/ anti-human nuclei / GFAP
ZH411
150 µm
a1
20 um
a2
20 um
Calbindin/ anti-human nucleui / EdU
a3
B
Calbindin/ anti-human nuclei / GFAP
ZH561
100 µm
b1
40 um
b2
30 um
Calbindin/ anti-human nuclei / EdU
b3

Figure S1 Proliferation and dissemination of human glioblastoma cells in brain slices. Spheroids of ZH411 (A) and ZH561 (B) tumour cells derived from primary tumour material were implanted in DIV 30 brain slices. Stacks of confocal IFA sections of brain slice around implanted tumour cells are shown at day 5 after implantation. a1, a2, b1 and b2 are higher magnification images of single sections of the compressed image stacks shown. a3 and b3 show EdU-positive nuclei.
